# LHX1 as a potential biomarker regulates EMT induction and cellular behaviors in uterine corpus endometrial carcinoma

**DOI:** 10.1016/j.clinsp.2022.100103

**Published:** 2022-09-15

**Authors:** Ye Tian, Fang Wen, Shuo Wang, Na Lv

**Affiliations:** a Department of Gynecology, Liaoning Cancer Hospital, Shenyang, China; b Department of Gynecology, The First Hospital, China Medical University, Shenyang, China; c Blood Collection Center, The First Hospital of China Medical University, Shenyang, China

**Keywords:** LHX1, Uterine corpus endometrial carcinoma, Prognosis, EMT induction, Bioinformatics analysis

## Abstract

**Objectives:**

To investigate the expression of LHX1 and its role as a biomarker in the diagnosis and prognosis of Uterine Corpus Endometrial Carcinoma (UCEC).

**Methods:**

The Cancer Genome Atlas (TCGA) database was used to detect the expression level of LHX1 in UCEC cells and tissues, and to find out the effect of LHX1 on prognosis. Co-expressed genes were then identified by Spearman correlation analysis, and the protein-protein interaction network was constructed using Cytoscape software. The R “clusterProfiler” package was used to conduct Gene Ontology (GO) and Kyoto Encyclopedia of Genes and Genomes (KEGG) pathway enrichment analyses. A series of *in vitro* experiments were performed to evaluate LHX1 expression and detect UCEC cell proliferation, invasion, and migration. Western blotting was used to determine the effect of LHX1 on expression levels of Epithelial-Mesenchymal Transition (EMT)-related proteins.

**Results:**

LHX1 was upregulated in UCEC tissues and correlated with poor overall survival and disease-specific survival outcomes. Functional enrichment analysis suggested that genes co-expressed with LHX1 were enriched in cell adhesion. The expression of LHX1 was positively correlated with the expression levels of genes related to EMT induction and invasion. LHX1 can enhance the proliferation, migration, and invasion activities of UCEC cells *in vitro*, and alter the expression levels of EMT-related proteins.

**Conclusion:**

LHX1 expression was highly upregulated in UCEC cells and tissues, which was correlated with the prognosis of patients with UCEC. LHX1 may regulate UCEC progression at least in part by modulating EMT induction.

## Highlights

• LHX1 is highly upregulated in the cells and tissues of Uterine Corpus Endometrial Carcinoma (UCEC).
• Upregulation of LHX1 is correlated with poor prognosis of UCEC patients.
• LHX1 may regulate UCEC progression at least partly by modulating EMT induction.

## Introduction

Uterine corpus cancer is the second most prevalent type of gynecological malignancy and the sixth most common type of cancer affecting women.^1^ In 2021, 66,570 new cases of uterine corpus cancer and 12,940 uterine corpus cancer-related deaths were reported worldwide.^1^ The incidence of endometrial cancer is significantly higher in developed countries (5.9%) compared with that in developing countries (4.0%),^2^ and the number of patients with endometrial cancer in the USA has been predicted to rise to 42.13 per 10,000 persons by 2030.^3^^,^^4^ Despite the advances in the targeted treatment for patients with Uterine Corpus Endometrial Carcinoma (UCEC), morbidity and mortality continue to rise.^1^, ^2^, ^3^ Although UCEC is frequently detected in earlier stages and is thus generally associated with a favorable prognosis, 10‒15% of patients ultimately develop metastasis that spreads beyond the regional lymph nodes and pelvis.^5^ Female patients exhibit a relatively poor prognosis, accounting for a disproportionate fraction of endometrial cancer-related morality.^6^ For patients with stage IV UCEC, the 5-year survival rate is estimated to range from 12% to 48%.^7^ Therefore, novel diagnostic/prognostic biomarkers and therapeutic targets for UCEC are essential to guide patient management.

LIM Homeobox 1 (LHX1) is a nuclear transcription factor in the LIM Homeodomain (LIM-HD) family, which consists of key transcriptional regulators that control organogenesis during embryonic development.^8^, ^9^, ^10^, ^11^ LHX1 expression is initially detected during gastrulation where it regulates cellular motility, and it is subsequently identified in the intermediate and lateral mesoderm. It has been reported to play an important role as a regulator of cell processes and cytoskeletal organization, as well as oncogenesis.^12^, ^13^, ^14^ Reports of LHX1 expression in endometrial tissues from both neonatal/adult mice and humans suggested that this transcription factor may also be an important regulator of endometrial development or remodeling.^11^ Some studies have demonstrated that LHX1 is expressed in diverse types of cancer cells, including leukemia, renal carcinoma, and breast cancer cells, in addition to epithelial cells.^14^, ^15^, ^16^, ^17^ For example, LHX1 overexpression has been reported in clear cell renal cell carcinoma, chronic leukemia and pancreatic cancer tissues,^18^, ^19^, ^20^ and its activation has also been detected in nephroblastoma and medulloblastoma tissues.^8^^,^^21^ There is evidence that LHX1 is a susceptibility gene in hepatitis B infection-associated hepatocellular carcinoma.^22^ The functional role of LHX1 in UCEC, however, has not yet been clarified.

In this study, the authors explored the role of LHX1 in UCEC and found that it was overexpressed in UCEC tissues and was significantly associated with poorer Overall Survival (OS) and Disease-Specific Survival (DSS). Using functional network analyses, it was revealed that the expression level of LHX1 was associated with cellular adhesion and positively correlated with the expression levels of key genes related to induction and invasion of Epithelial-Mesenchymal Transition (EMT). *In vitro* analyses further indicated that LHX1 could regulate migratory, invasive, and proliferative capabilities of UCEC cells, and alter expression patterns of EMT-related proteins. Consequently, these data might provide preliminary insight into the role of LHX1 in UCEC cells and tissues and facilitate further research on the targeted treatment of patients with UCEC.

## Materials and methods

### Data collection

The UCSC Xena database (https://xenabrowser.net/datapages/) was used to download clinical and RNA-seq data in Transcripts Per Million reads (TPM) format from Genotype-Tissue Expression (GTEx) and the Cancer Genome Atlas (TCGA) databases. The RNAseq data were log2-transformed, and LHX1 expression in UCEC tissues and normal tissues was detected.

### Data preprocessing

R “affy” package was used to analyze the original probe-level data in CEL files by Robust Multi-array average Algorithm (RMA), and then quantile normalization and correction for the background were performed to obtain gene expression data.^23^ The average expression value for genes with multiple probes was calculated.^24^

### Prognostic analysis

The association of LHX1 expression with OS and DFS of UCEC patients was predicted through Kaplan-Meier and log-rank tests using the survival package in R software,^25^ with *p* < 0.05 as the significance threshold. Patients were divided into two groups (LHX1 high expression group and LHX1 low expression group) according to the median LHX1 expression level, and survival outcomes between the two groups were compared.

### Functional enrichment analysis

The R “clusterProfiler” package was used for the classification and enrichment analysis of gene clusters,^23^ and Gene Ontology (GO) terms and Kyoto Encyclopedia of Genes and Genomes (KEGG) pathways enrichment analyses were conducted to assess the genes co-expressed with LHX1. The p-value threshold was adjusted to 0.05 for the terms with significant enrichment.

### Cell culture and transfection

Human Endometrial Stromal Cells (hESCs) and Ishikawa and HEC-1B UCEC cell lines were obtained from the American Type Culture Collection (ATCC; Manassas, VA, USA). Cells were cultured in a Dulbecco's modified Eagle's medium (DMDM; Gibco, New York, NY, USA) containing 10% fetal bovine serum (FBS; Thermo Fisher Scientific, Inc., Waltham, MA, USA) and penicillin/streptomycin at 37°C in a humidified 5% CO_2_ incubator.

Cellular transfection was performed using Lipofectamine 2000 (Invitrogen, Carlsbad, CA, USA) when cells reached a confluence of 80%, in which Ishikawa cells were transfected with short hairpin RNAs (shRNAs; sh1-LHX1 and sh1-LHX2), while HEC-1B cells were transfected with pcDNA3.1-LHX1 or empty vector control. The target sequences of shRNAs were as follows: sh1-LHX1: 5ʹ- GAACGACTTCTTCCGGTGTTT-3ʹ, sh2-LHX1: 5ʹ- CGTCCAGTGCTGTGAATGTAA -3ʹ.

### RNA extraction and quantitative reverse transcription-polymerase chain reaction (RT-qPCR) analysis

After transfection, RNA was extracted from cells using TRIzol reagent (Invitrogen), and cDNA was then prepared using the SuperScript™ III with PlatinumTM Taq High Fidelity DNA polymerase (Invitrogen). The LHX1 expression level was detected using the FastStart Universal SYBR Green Master (Roche, Basel, Switzerland) with an ABI 7500 system. The primers used for RT-qPCR were as follows: LHX1: F:5ʹ-CCTGGACCGCTTTCTCTTGAA-3ʹ, R:5ʹ-ACCGAAACACCGGAAGAAGTC-3ʹ; GAPDH: F:5ʹ-CTCACCGGATGCACCAATGTT-3ʹ, R:5ʹ- CGCGTTGCTCACAATGTTCAT-3ʹ.

Thermocycling conditions were as follows: at 95°C for 5 min; 30 cycles at 95°C for 30 s, at 60°C for 45 s, at 72°C for 30 s; and at 72°C for 5 min. All experiments were performed in triplicate, and the relative gene expression levels were calculated using the 2^−ΔΔCt^ method.

### Western blot analysis

RIPA buffer (Cell Signaling Technology [CST], Danvers, MA, US) was used to lyse cells, and the extracted proteins were quantified via a BCA Protein Assay kit (CST). A total of 30 µg of each sample was then separated using sodium dodecyl-sulfate polyacrylamide gel electrophoresis (SDS-PAGE; 90 min, 120 V, 60 mA), followed by transferring onto Polyvinylidene Difluoride (PVDF) membranes (Sangon Biotech Co., Ltd., Shanghai, China). Blots were blocked with 5% non-fat milk (Sangon Biotech Co., Ltd.) and incubated overnight with appropriate primary antibodies at 4°C. Glyceraldehyde 3-Phosphate Dehydrogenase (GAPDH) served as a loading control. Afterwards, the membranes were incubated with primary antibodies (anti-E-cadherin (CST, #3195, 1:1000, Rabbit mAb), anti-N-cadherin (CST, #13116, 1:1000, Rabbit mAb), anti-vimentin (CST, #5741, 1:1000, Rabbit mAb), anti-Snail (CST, #3879, 1:1000, Rabbit mAb), and anti-Slug (CST, #9585S, 1:1,000, Rabbit mAb) antibodies) at 4°C overnight using GAPDH (CST, #5174, 1:1000, Rabbit mAb) as an internal reference gene. Blots were then probed with the secondary Horseradish Peroxidase (HRP)-conjugated anti-rabbit IgG antibody (CST, #7074, 1:3000) for 1h at room temperature. Protein bands were subsequently detected using the SignalFire™ ECL Reagent (CST), and the ImageLab software (ver. 4.1; Bio-Rad Laboratories Inc., Hercules, CA, USA) was used for the subsequent data analysis.

### Cell counting kit-8 (CCK-8) assay

24 h after transfection, cells were seeded onto 96-well plates (1000 cells/well). After 0 h, 24 h, 48 h, and 72 h, 10 µL ccK-8 reagent was added to each well and cultured at 37°C for another 2 h to assess cell proliferation. The absorbance was recorded at 450 nm using a microplate reader and the proliferation curve was plotted.

### Colony formation assay

UCEC cells were suspended in a DMEM containing 10% FBS at 1000 cells/mL and added to 6 cm culture plates. After 2 weeks of incubation, cells were rinsed three times with phosphate-buffered saline, fixed using 4% paraformaldehyde, and stained with Giemsa for 20 min. Visible colonies composed of > 50 cells were counted under a microscope (Leica, Wetzlar, Germany).

### Wound healing assay

Cell migration was assessed via wound healing assay as reported previously.^26^ Briefly, cells were grown to reach a confluence of 30‒50% in a 6-well plate, with a straight scratch wound that was generated in the monolayer surface using a 20 μL pipette tip. An Olympus 1×71 camera system (Olympus, Tokyo, Japan) was then used to image the closure of the generated wound at 0 and 24 h, respectively.

### Transwell analyses

Transwell inserts (Corning Inc., Corning, NY, USA) were used to assess the invasion and migration of UCEC cells. For invasion assays, Transwell chambers were pre-coated with Matrigel (BD Biosciences, Franklin Lakes, NJ, USA) and incubated for 4h at 37°C. 24 h after transfection, a total of 5000 or 10,000 cells in serum-free media were added to the upper chamber of each Transwell to analyze migration and invasion, respectively. The lower chamber was filled with 500 μL of media containing 10% FBS. Following 24-h incubation, cotton swabs were used to remove non-migratory/invasive cells, while the remaining cells were fixed, stained for 30 min, and imaged by microscopy (Olympus).

### Statistical analysis

The frequency and mRNA expression levels of LHX1 were calculated. Descriptive analyses of normality and homogeneity of variance were conducted for continuous variables. Relationships between specific variables and LHX1 expression levels were analyzed via the *t*-test, Mann-Whitney *U* test, Kruskal-Wallis test, or Analysis of Variance (ANOVA), as appropriate. These analyses and the Receiver Operating Characteristic (ROC) curve were generated using GraphPad Prism 5.0 software (GraphPad Software Inc., San Diego, CA, USA). Survival analysis was conducted by Kaplan-Meier curves and the log-rank test with a 95% Confidence Interval (95% CI) using R software;^27^ *p* < 0.05 was considered statistically significant. Variables with *p* < 0.2 in univariate analysis were retained for multivariate logistic regression analysis, and a stepwise forward Cox regression approach was used for further analysis.

## Results

### LHX1 expression was a diagnostic biomarker for UCEC

**Fig. 1:**
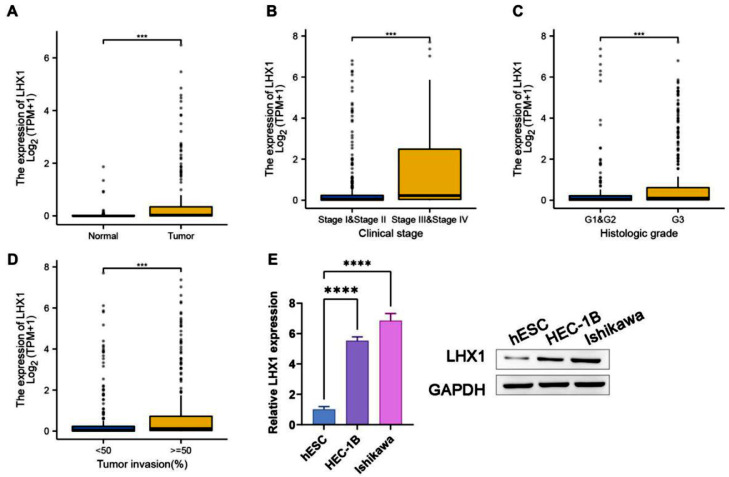
LHX1 upregulation in UCEC tissues. (A) Comparison of LHX1 expression levels in UCEC tissues and healthy tissues using data obtained from TCGA database (Match TCGA normal and GTEx data, *p* < 0.001). Individual points correspond to specific samples. (B) Comparison of relative LHX1 expression levels in patients with stage I/II or stage III/IV UCEC. (C) Comparison of LHX1 expression levels in patients with histological stage G1/G2 or G3 UCEC. (D) Relative LHX1 expression levels in UCEC tissues were significantly associated with the depth of tumor invasion. (E) RT-qPCR and Western blotting were used to assess LHX1 expression levels in different cell lines. Results are expressed as mean ± SD; *p < 0.05, **p < 0.01, ***p < 0.001. Data are representative of three independent experiments.

To explore the effect of LHX1 expression on UCEC, the authors downloaded gene expression data from TCGA database and compared LHX1 expression levels in UCEC tissues and adjacent healthy endometrial tissues. LHX1 expression levels were higher in UCEC tissues than in adjacent healthy endometrial tissues (Fig. 1A). Besides, LHX1 was found to be expressed at higher levels in tumors from patients with stage III/IV disease compared with stage I/II disease (Fig. 1B). The LHX1 expression level was also found to be higher upon loss of histological differentiation, indicating that the LHX1 expression level was significantly higher in G3 tumors compared with G1/G2 tumors (Fig. 1C). In UCEC patients, the LHX1 expression level was also positively correlated with the depth of tumor invasion (Fig. 1D). When LHX1 mRNA levels were compared between UCEC cells and control hESCs via RT-qPCR and Western blotting, they were found to be significantly upregulated in UCEC cells (p < 0.00001; Fig. 1E). Therefore, LHX1 can be regarded a diagnostic biomarker for UCEC.

### Upregulation of LHX1 expression levels was correlated with a poorer prognosis of UCEC patients

**Fig. 2:**
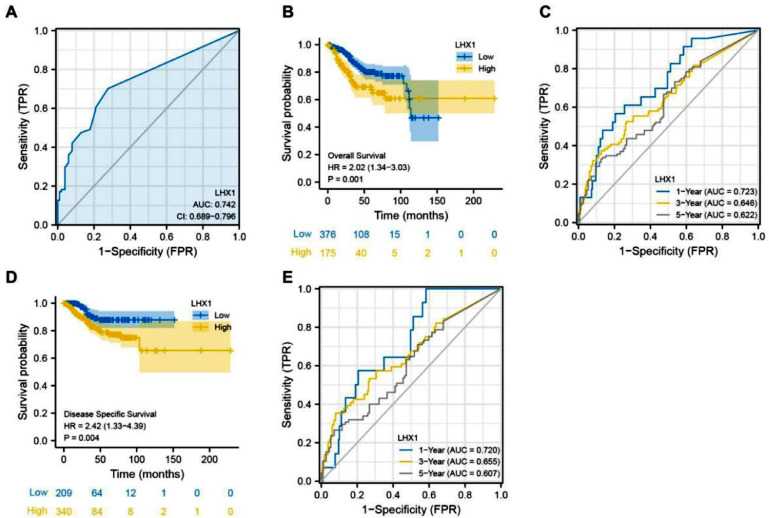
LHX1 upregulation is associated with poorer prognostic outcomes of UCEC patients. (A) AUC curves were plotted to assess the relevance of LHX1 expression levels within TCGA cohort. (B) Kaplan-Meier analyses were used to explore the relationship between LHX1 expression levels and Overall Survival (OS) of UCEC patients. (C) Time-dependent ROC curves were used to assess the 1-, 3-, and 5-year survival rates of UCEC patients. (D) The relationship between LHX1 expression levels and disease-specific survival (DSS) of UCEC patients was assessed by Kaplan-Meier curves. (E) Time-dependent ROC curves were used to assess the 1-, 3-, and 5-year DSS of UCEC patients.

Survival analysis was conducted to assess the association of LHX1 expression levels with a prognosis of UCEC patients. ROC curves indicated that LHX1 expression levels could be utilized to diagnose UCEC patients with high sensitivity (Area Under the Curve ‒ AUC = 0.742, Fig. 2A), while Kaplan-Meier curves showed that the reduced LHX1 expression level was correlated with worse OS of UCEC patients (Fig. 2B). The AUC values for 1-, 3-, and 5-year survival rates in time-dependent ROC curve analyses were 0.723, 0.646, and 0.622, respectively (Fig. 2C), supporting the potential of this gene as an independent predictor of patient prognosis. LHX1 expression levels were also significantly associated with DSS of UCEC patients (Fig. 2D), with AUC values of 0.720, 0.655, and 0.607 for 1-, 3-, and 5-year DSS, respectively (Fig. 2E). These results suggested that LHX1 expression levels could be used a prognostic biomarker for UCEC.

### Analysis of the functional roles of genes co-expressed with LHX1 in UCEC

**Fig. 3:**
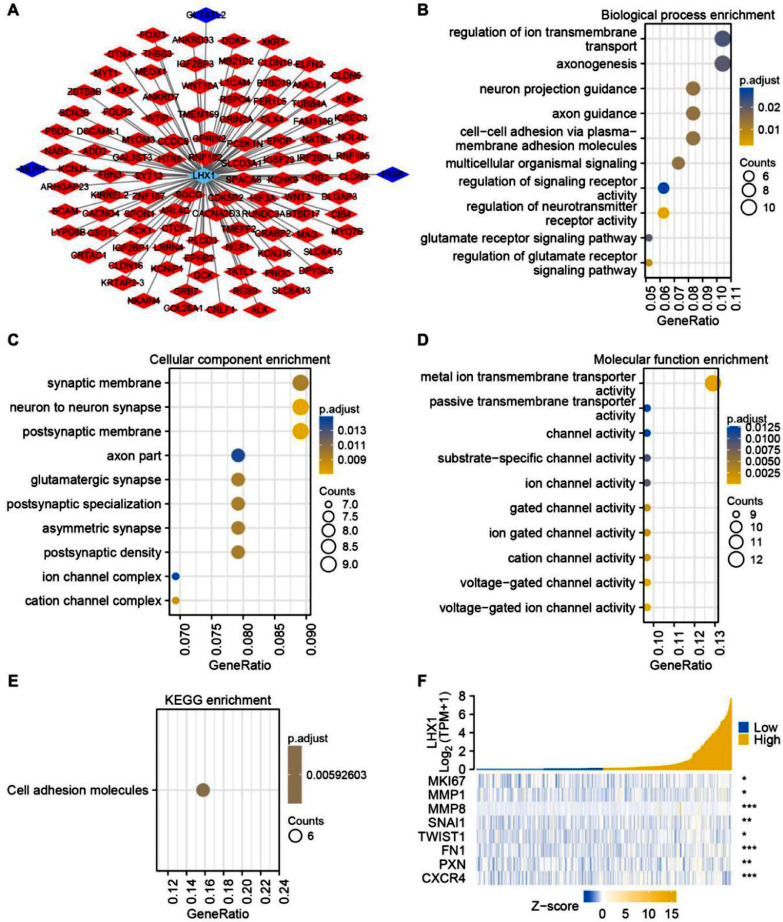
Analysis of genes co-expressed with LHX1 in UCEC. (A) Genes co-expressed with LHX1 in UCEC were identified and visualized through Cytoscape software. Nodes in red and blue respectively corresponded to positively correlated genes and negatively correlated genes. (B) Biological process terms. (C) Cellular component terms. (D) Molecular function terms. (E) KEGG pathway analysis. (F) A positive correlation was observed between LHX1 expression levels and expression levels of genes related to the EMT and invasion. Altered genes are noted with a red stare, while upregulated and downregulated genes are respectively denoted in yellow and blue.

To more fully understand how LHX1 expression levels can influence UCEC cell development or malignancy, Spearman correlation analysis was used to identify genes co-expressed with LHX1. In total, 102 and 3 genes were respectively found to be positively and negatively correlated with LHX1 expression levels significantly (*p* < 0.05) (Fig. 3A). The GO functional analysis of these co-expressed genes revealed that they were enriched in biological processes, including regulation of neurotransmitter receptor activity and multicellular organismal signaling (Fig. 3B), cellular component terms, such as neuron-to-neuron synapse and postsynaptic membrane (Fig. 3C), and molecular function terms, including various channel activities and metal ion transmembrane transporter activity (Fig. 3D). The KEGG pathway analysis additionally indicated the enrichment of these genes in pathways associated with cellular adhesion (Fig. 3E). The expression association between LHX1 and invasion- and EMT-associated genes was further assessed through Spearman correlation analysis, revealing a strongly positive correlation between LHX1 and SNAI1, MMP8, and CXCR4 (Fig. 3F). Collectively, these data supported the role of LHX1 expression levels as a regulator of cellular adhesion and EMT induction.

### Overexpression of LHX1 enhanced UCEC cell proliferation, invasion, migration, and EMT induction

**Fig. 4:**
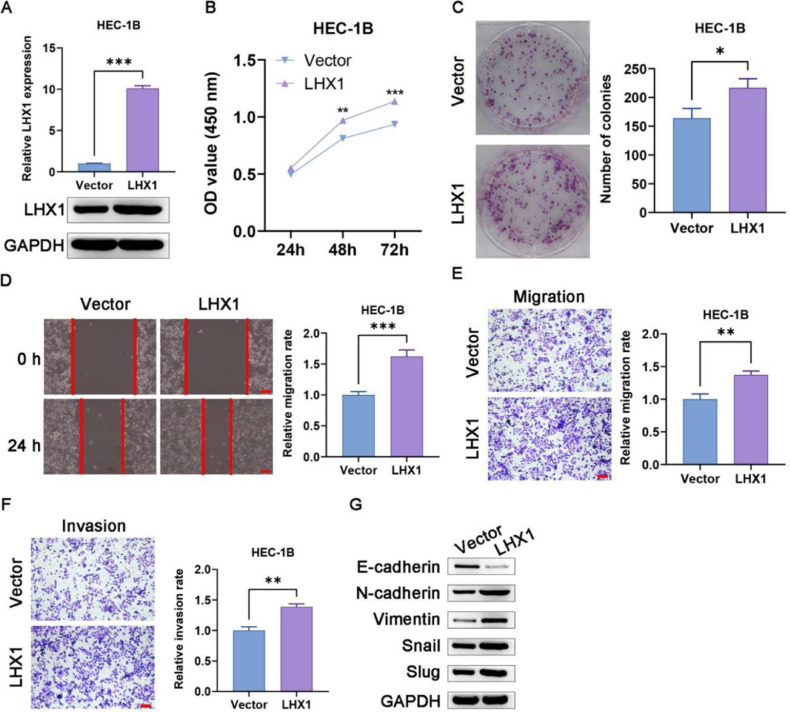
LHX1 overexpression enhances UCEC cell migration, invasion, proliferation, and EMT induction. (A) LHX1 expression was confirmed to be increased via RT-qPCR and Western blotting compared with that in cells treated with the empty pcDNA3.1 vector. (B) CCK-8 assay was used to analyze cellular proliferation. (C) Overexpressed LHX1 enhanced the colony formation capability of HEC-1B cells compared with empty vector transfection. (D) The influence of LHX1 overexpression on HEC-1B cell migration was examined via wound healing assay. (×100; scale bar, 100 μm) (E) Cellular migration and (F) invasion were analyzed and quantified. (×100; scale bar, 100 μm) (G) LHX1 overexpression enhanced the expression of Slug, Snail, Vimentin, and N-cadherin, while inhibited the expression of E-cadherin. Results are expressed as mean ± SD; *p < 0.05, **p < 0.01, ***p < 0.001. Data are representative of three independent experiments.

According to the results of RT-qPCR and Western blotting, pcDNA3.1-LHX1 transfection was confirmed to enhance LHX1 expression in HEC-1B cells (Fig. 4A). Such overexpression markedly increased the proliferation of these cells in CCK-8 and colony formation assays compared with pcDNA3.1 empty vector transfection (Figs. 4B, C). The exogenous upregulation of LHX1 expression levels additionally enhanced migration of HEC-1B cells in wound healing assay (Fig. 4D), and augmented invasion and migration in Transwell assays compared with pcDNA3.1 empty vector transfection (Figs. 4E, F). Given the critical role of EMT induction in tumor progression, the authors also assessed the expression levels of EMT-associated genes in these cells and observed significantly reduced expression of E-cadherin following LHX1 overexpression and a concomitant rise in expression levels of Slug, Snail, Vimentin, and N-cadherin (Fig. 4G). Taken together, the above-mentioned results demonstrated that LHX1 expression enhanced UCEC cell malignancy and EMT induction.

### Knockdown of LHX1 suppressed UCEC cell invasion, migration, proliferation, and EMT induction

**Fig. 5:**
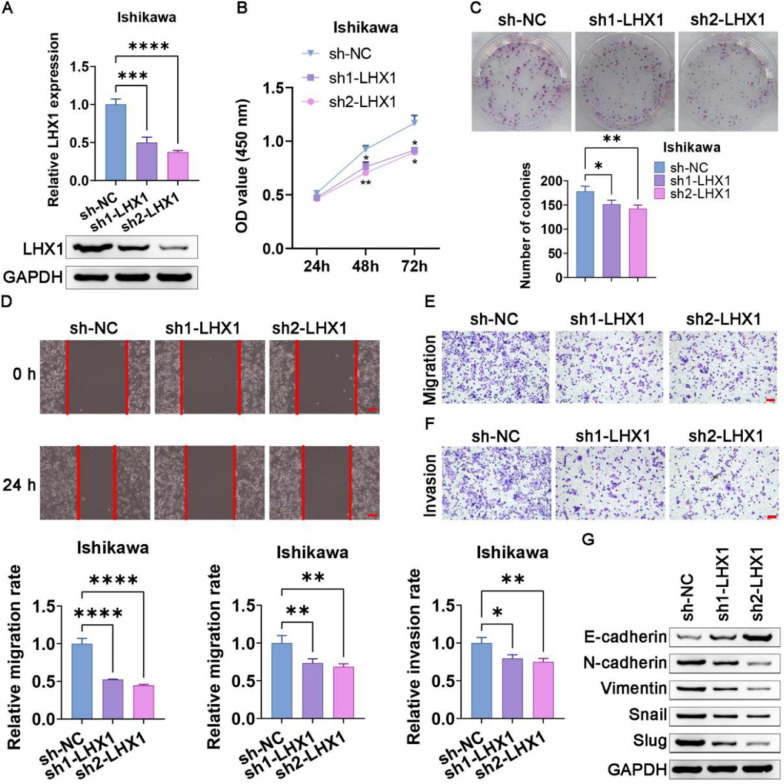
LHX1 knockdown suppresses UCEC cell migration, proliferation, invasion, and EMT induction. (A) LHX1 expression was markedly reduced in RT-qPCR and Western blot assays following sh1-LHX1/sh2-LHX1 transfection. (B) The CCK-8 assay was used to assess cellular proliferation. (C) The colony formation capability of Ishikawa cells was reduced following LHX1 knockdown relative to sh-NC transfection. (D) Ishikawa cell migration decreased in the wound healing assay following LHX1 knockdown (×100; scale bar, 100 μm). (E) Cellular migration and (F) invasion were analyzed and quantified. (G) LHX1 knockdown enhanced E-cadherin expression, while reduced the expression levels of Snail, Slug, Vimentin, and N-cadherin. Results are expressed as mean ± SD; *p < 0.05, **p < 0.01, ***p < 0.001. Data are representative of three independent experiments.

When Ishikawa cells were transfected with the sh1-LHX1 and sh2-LHX1 constructs, a successful downregulation of LHX1 was confirmed via RT-qPCR and Western blotting (Fig. 5A). CCK-8 and colony formation assays subsequently revealed that LHX1 knockdown significantly impaired the proliferation of Ishikawa cells compared with sh-NC transfection (Fig. 5 B, C). Consistently, LHX1 knockdown impaired the ability of Ishikawa cells to migrate in the wound healing assay (Fig. 5D), in addition, to suppress migration and invasion in the Transwell assay compared with sh-NC treatment (Fig. 5 E, F). Consistently, these results suggested that the knockdown of LHX1 suppressed proliferation, migration, and invasion of Ishikawa cells. Western blotting additionally indicated that E-cadherin expression was elevated following LHX1 knockdown, whereas the expression levels of Slug, Snail, Vimentin, and N-cadherin were reduced (Fig. 5G). Collectively, these data confirmed the ability of LHX1 to modulate the expression levels of EMT-associated proteins in UCEC cells.

## Discussion

Currently, UCEC is one of the most common gynecological malignancies and the sixth most prevalent type of cancer affecting women globally.^1^ The incidence rate of UCEC continues to rise with changes in lifestyle and life expectancy, and the incidence rate has noticeably increased in younger women over the last 20 years.^28^ While the mechanisms governing UCEC incidence have still remained elusive, genetic factors, obesity and drug use are all thought to be involved.^29^^,^^30^ Therapeutic efficacy can be achieved in less than 95% of patients with early-stage UCEC.^31^ Although serum biomarkers including carbohydrate antigen-19-9, carbohydrate antigen-125, and carcinoembryonic antigen have been shown to play a role in the diagnosis of UCEC, they were upregulated in only 20‒30% of patients with UCEC.^22^^,^^32^ When UCEC diagnosis is delayed, therapeutic options are limited and patients are at a higher risk of postoperative recurrence or metastasis, both of which are associated with poorer prognostic outcomes.^33^ The expression of certain genes that are frequently dysregulated in patients with UCEC can serve as diagnostic biomarkers.^34^ Consequently, further research is essential to explore more potential biomarkers to guide clinicians in the faster diagnosis, prognostic evaluation, and treatment of UCEC patients.

LHX1 was initially found to play a role in the functions of renal and brain tissues.^35^ More recent evidence, however, has also shown that it was overexpressed or reactivated in certain types of cancer.^14^^,^^36^ In the present study, the authors found that LHX1 was upregulated in UCEC tissues compared with healthy tissues, which was significantly associated with poorer OS and DFS outcomes. This is consistent with prior evidence, indicating that LHX1 can be regarded as a tumor cell marker.^14^

In light of the above-mentioned evidence, the authors conducted functional assays using HEC-1B and Ishikawa cells, confirming the oncogenic role of LHX1 in UCEC. EMT induction is a key stage in the process of metastatic progression that alters the adhesion, migration, and invasion of malignant cells.^37^^,^^38^ Reduced E-cadherin expression is a fundamental stage in this process,^39^ and the authors found that LHX1 knockdown was associated with increased E-cadherin expression, whereas the opposite outcome was observed in HEC-1B cells when LHX1 was overexpressed. Bioinformatics-based enrichment analyses suggested that changes in LHX1 expression levels could regulate cellular adhesion and EMT induction, demonstrating that LHX1 could control the EMT process at least in part via regulating E-cadherin expression levels, which is consistent with the ability of mesenchymal transcription factors to inhibit E-cadherin expression and promote progression of the EMT.^40^ In the present study, it was found that expression levels of Slug, Snail, Vimentin, and N-cadherin were higher in cells overexpressing LHX1, which is in line with the ability of LHX1 to regulate UCEC progression at least in part via modulation of the EMT process.

These results suggested new insights into the mechanistic relationship between LHX1 expression levels and UCEC malignancy, but there are also certain limitations. First, the authors only conducted *in vitro* mechanistic studies, and further *in vivo* validation is therefore required. In addition, several other signaling pathways play an interrelated role in the coordination of EMT induction, including the Wnt/beta-catenin and Akt/PI3K signaling pathways.^41^^,^^42^ More detailed insights regarding the role of LHX1 in UCEC need to be further investigated by more studies.

In summary, it was found that LHX1 expression levels were highly upregulated in UCEC cells and tissues, which was associated with a decrease in OS. The oncogenic role of LHX1 expression levels was further confirmed through knockout and overexpression studies, which revealed that it enhanced the proliferative, invasive, and migratory capabilities of UCEC cells while regulating the expression levels of key EMT-associated proteins, including Slug, Snail, Vimentin, E-cadherin, and N-cadherin. LHX1 expression levels may thus be a prognostic biomarker and a promising therapeutic target for UCEC patients.

## Authors' contributions

Ye Tian and Fang Wen designed experiments. Fang Wen, Shuo Wang and Na Lv carried out experiments and analyzed experimental results. Ye Tian wrote the manuscript, Ye Tian and Fang Wen revised the manuscript. All authors approved the final manuscript.

## Funding

This research did not receive any specific grant from funding agencies in the public, commercial, or not-for-profit sectors.

## Declaration of Competing Interest

The authors declare no conflicts of interest.
